# Educators’ occupational well-being in health and social care education

**DOI:** 10.1093/occmed/kqac024

**Published:** 2022-05-05

**Authors:** J Rinne, H Leino-Kilpi, T Saaranen, M Pasanen, L Salminen

**Affiliations:** Department of Nursing Science, University of Turku, Turun Yliopisto, Finland; Department of Nursing Science, University of Turku, Turun Yliopisto, Finland; Turku University Hospital, Turku, Finland; Department of Nursing Science, University of Eastern Finland, Kuopio, Finland; Department of Nursing Science, University of Turku, Turun Yliopisto, Finland; Department of Nursing Science, University of Turku, Turun Yliopisto, Finland; Turku University Hospital, Turku, Finland

**Keywords:** Educator, occupational health, occupational well-being, resources, workload

## Abstract

**Background:**

The occupational well-being (OW) of educators can be defined as a balance between resources and workload factors as seen from four aspects of working life: (i) individual, (ii) working conditions, (iii) professional competence and (iv) work community. The research in this study examined the individual aspect as particular importance to the physical and mental workability of educators.

**Aims:**

To study the individual aspect of the OW of educators as well as the associating factors.

**Methods:**

A cross-sectional survey design was conducted among educators working in health and social care education in Finland. The data were collected with an electronic survey using the ‘Occupational well-being of social and health care teachers—index questionnaire’. The data were analysed with an SPSS version 27 using descriptive statistics, explorative factor analysis and linear regression analysis.

**Results:**

The educators (*n* = 552, response rate 31%) assessed their resources for managing their mental workload as quite poor (2.41, standard deviation [SD] 0.98). In addition, workplace support promoting OW was assessed as being quite poor (2.37, SD 0.88), and as especially requiring more measures during working hours. Associations with the individual aspect of OW were found between the personal and work-related background variables as well as overall OW.

**Conclusions:**

The perceptions of the educators indicated that resources to cope with workload factors should be promoted. Investing in educators’ resources at work, enabling well-being actions during working hours and avoiding backlog situations would all help promote the educators’ OW.

**Key learning points**

What is already known about this subject:

- Educators working in health and social care have high levels of mental workload.
- Heavy workloads are becoming a challenge regarding personal resources at work.

What this study adds:

- The individual aspect of occupational well-being for educators is associated with an improvement in overall occupational well-being.
- From the perspective of the educators, the personal and communal resources provided to manage educators’ mental workload factors (e.g. backlog situations and having no time for breaks) are currently quite poor.
- The more experienced educators expressed more dissatisfaction with the resources available for mental workload factors.

What impact this may have on practice or policy:

- Investing in promoting resources that will help educators to manage workload factors in their daily work needs to be recognized as being associated with their overall occupational well-being.
- Workplace support is needed to enhance occupational well-being by promoting activities during working hours and avoiding backlog situations at work.
- Further intervention studies are needed to find feasible ways to promote personal resources, especially during working hours.

## Introduction

Occupational well-being (OW) is important to both the educators and their organizations as it positively affects their work productivity [1] and the quality of education provided [2]. The work of educators is psychosocially demanding, as it includes not only the demands of students but also a heavy workload, both of which create challenges when maintaining a well-being balance between personal and working life [3]. Internationally, the Health and Social Care Education (HSCE) workforce is ageing, and the heavy workloads are not only becoming a challenge for workability but a problem as regards attracting new educators into the field [4,5]. There is also constant pressure to educate more students, especially those in nursing care due to the nursing shortage in many countries [6,7].

**Figure 1.**
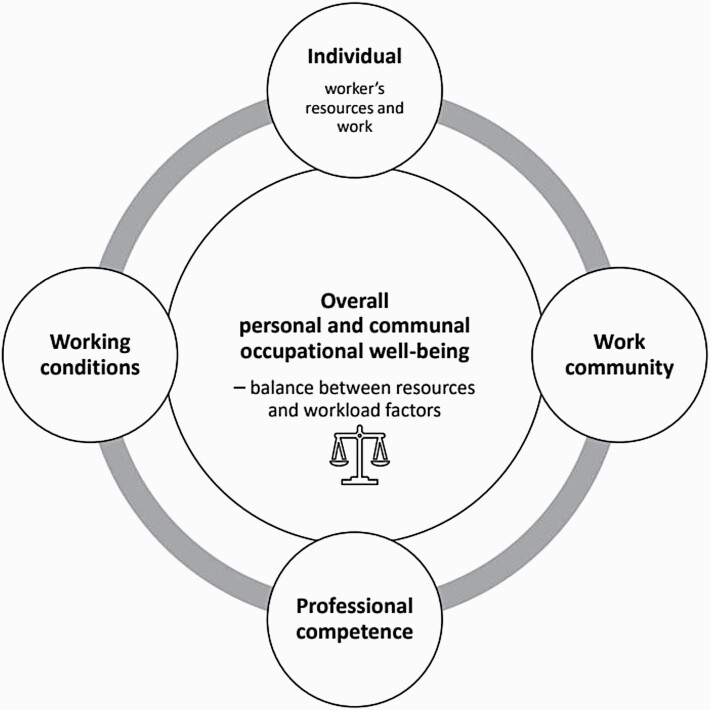
The aspects of educators’ overall personal and communal OW (modified from OWSS Model [9).

The definition of OW varies within different fields of research. The International Labour Organization [8] considers OW as being constructed from all aspects of working life, including the workers’ perceptions of their work, the physical and mental working environment and the working community; considering these aspects will generate healthy, satisfied and engaged workers. Similar aspects are found in the model used in this study, The “Content Model for the Promotion of School Community Staff’s Occupational Well-being” (OWSS) [9]. In this model, the overall OW of the educators is defined as the balance between resources and workload factors as expressed in four aspects of working life (Figure 1). The focus of this study is on one of the four aspects in this model, the aspect of the individual, and thus considers the educators’ personal resources, health, fitness and vigour to manage workload factors and communal support. There has been relatively little OW research conducted from this point of view. In previous studies, the focus has been more on the educators’ satisfaction with working conditions, their salary and job description [10,11].

This study addresses the personal and communal resources of the individual when managing physical and mental workload factors; the study does not consider either the educator’s role in the organization or their job description. The personal resources, health, fitness and vigour, of educators can be defined as the individual’s ability to perform daily activities with optimal performance, endurance, strength and energy and to be able to manage the disease, fatigue or stress [12–14]. The mental strain of a heavy workload and student demands was found to be negatively associated with OW [3,10]. Physical strain, such as the necessity for prolonged sitting times, was associated with health issues, such as diabetes, hypertension and hypercholesterolaemia [15].

From the aspect of the individual, the communal resources are the support provided by the workplace and occupational health care services for the educators. Workplace support, such as respect and understanding from the management for the educators’ work demands and the support provided regarding workload factors, is insufficient in previous studies; this deficiency affects the well-being of educators [3,16]. Occupational health care services, the main purpose of which is to maintain and promote worker resources, are insufficient in the working population from a global perspective [17]; in the context of this study, there was a lack of knowledge about the support required.

Personal and work-related factors are associated with OW among educators working in HSCE. The work–life balance has proved to be more problematic among women and as having a greater effect on their OW [10,16]. Age and work experience have been found to have associations with OW as older educators are more willing to leave their current position [10]. A sense of belonging to a working community is also connected with affective feelings towards work; however, this is the most problematic aspect among the less experienced nurse academics and consequently detrimental to their OW [3,16]. Clarity, as regards tenure and work autonomy, is associated positively with well-being at work [3,10].

Overall, as the individual aspect of educators working in HSCE is quite complex it necessitates more research. Additional information is needed regarding the personal resources required to deal with workload factors and the type of communal support necessary. In addition, there is a need to clarify the factors associated with the individual aspect of OW. The purpose of this study was to examine OW from the aspect/perspective of the individual educators working in HSCE. Two main research questions were formulated:

1) What is the individual aspect of OW as perceived by educators working in HSCE in Finland?
2) What personal, work-related and overall OW factors are associated with the individual aspect of OW among educators working in HSCE?

## Methods

The data for this cross-sectional descriptive survey design were gathered in September 2020 as part of the ‘Occupational Well-being of Social and Health Care Educators’—project in cooperation with two universities in Finland. Participants were recruited from the register of the Trade Union of Education in Finland, which is a labour union with a membership of ~70% of the total national educational workforce in HSCE. In Finland, educators with a permanent employment contract in HSCE are qualified professionals having a master’s degree from a university or a university of applied sciences (European Qualifications Framework, EQF 7). These qualifications include pedagogical studies for teaching general or vocational subjects to the future health and social care workforce. They mostly work in educational rather than clinical settings.

The research data were collected with an electronic questionnaire, ‘Occupational well-being of social and health care teachers—index questionnaire’, sent out by the Trade Union of Education in Finland to the eligible participants by e-mail; this was followed 3 weeks later by two reminders. The e-mail contained information about the study and a link to the questionnaire. This questionnaire has been used and developed previously by school staff for researching national and international basic education (i.e. [18]) and has also been developed further by educators working in the HSCE sector in Finland [19]. In this project, the research team made an additional development to the questionnaire (e.g. adding new questions, especially to the demographical section) and then piloted this version with educators working in HSCE (*n* = 33) in Spring 2020.

In this study, focusing on the individual aspect of OW, the 15-item subscale of the questionnaire the ‘Worker’s Resources and Work’ (WRW, Likert scale 1 = total disagreement to 5 = total agreement) is reported for statements that included questions such as ‘the mental workload of my work is appropriate’ and ‘I have time to take breaks and have moments of rest in my work’. In this section, three new questions were added to the previously used questionnaire [19], but no changes were made after this version was piloted in spring 2020. The WRW subscale is reported along with the background variables and the overall OW variables using the continuous scale (0 = very poor to 5 = very good) included in the ‘Occupational well-being of social and health care teachers—index questionnaire’ (Table 1).

**Table 1.** Educators’ personal, work and overall OW variables

Personal		*n* (%)	
Gender (*n* = 550)	Female	509 (92)	
	Male	36 (7)	
	Other/prefer not to say	5 (1)	
Age in years (*n* = 547)	39 ≥	56 (10)	
	40–49	161 (29)	
	50–59	227 (42)	
	60 ≤	103 (19)	
Marital status (*n* = 548)	Married/co-habiting/in registered partnership	422 (77)	
	In a relationship	39 (7)	
	Single/widowed	78 (14)	
	Other	9 (2)	
Underage child/children in the family (*n* = 548)	Yes	210 (38)	
	No	338 (62)	
Taking care of another person (not related to my job) needing help due to advanced age, illness or disability (*n* = 548)	Yes	193 (35)	
	No	355 (65)	
Work		*n* (%)	
Work includes remote working (*n* = 548)	Yes	308 (56)	
	No	240 (44)	
Work experience (years) as an educator in HSCE (*n* = 548)	10 >	181 (33)	
	10–20	255 (46)	
	20 <	112 (21)	
Current employment contract (*n* = 548)	Permanent	499 (91)	
	Temporary	49 (9)	
Another additional job (*n* = 547)	Yes	106 (19)	
	No	441 (81)	
Overall OW (interval scale 0–5, 0 = very poor to 5 = very good)		Mean	SD
Personal OW (*n* = 538)		3.19	1.14
Communal OW (*n* = 533)		2.61	0.96
Satisfaction with actions promoting OW in my working community (*n* = 495)		2.27	1.30
Satisfaction with actions promoting OW during leisure time (*n* = 522)		3.31	1.07

The study followed ethical guidelines [20]. Ethical approval was granted by the Ethical committee of the University of Eastern Finland (October 2020) and permission for the study before the data collection was granted in the spring of 2020 by the Trade Union of Education in Finland. The Trade Union distributed the link to the questionnaire via e-mail and the researchers were not able to access the respondent’s contact information. The participants were not able to proceed with the electronic questionnaire without checking the box at the beginning of the questionnaire giving their informed consent and acknowledging the privacy policy stated. Participation was voluntary and confidential, emphasis was placed on the participant’s anonymity and the research data were managed with high confidentiality according to general data protection regulation (GDPR) [21].

The data were analysed with an SPSS version 27 in three phases. First, the descriptive statistics, frequencies and percentages were calculated in each variable under research. Secondly, the explorative factor analysis (EFA) was used to identify the factor structure of the set variables in the WRW, because new questions had been added to the questionnaire. The EFA was conducted using the principal axis factoring extraction method (Promax). Bartlett’s test for sphericity and the Kaiser–Meyer–Olkin Measure for Sampling Adequacy found the data to be appropriate for the EFA [22]. Four new sum variables were extracted (sum variables, *n* = 527, Table 2) with the Kaiser rule. One item was omitted from the EFA due to low loading (0.26), communality (0.10) and cross-loading [22]. In this study, the satisfactory values for factor loadings were preferably above 0.40 (one loading < 0.4) and commonalities above 0.30 (0.36–0.82). Three items with low communality values (0.16–0.26) remained in the factor analysis due to reasonable loadings and being theoretically plausible [22]. The total variance explained by the extracted factors in the instrument was 52% and the Cronbach’s alpha (0.84) of the overall scale was good [23].

**Table 2.** The individual aspect of OW

		Mean	SD	Cronbach’s α
WRW (*n* = 527, 15 items)		2.78	0.67	0.84
Sum variable	Variable			
Resources and mental workload		2.41	0.98	0.85
	Appropriateness of mental workload	2.65	1.21	
	Satisfaction with workload	2.67	1.30	
	Backlog can be avoided	1.80	1.01	
	Time to take breaks and moments of rest	2.56	1.23	
Resources and physical workload		3.49	0.83	0.58
	Appropriateness of physical workload	3.92	0.98	
	Musculoskeletal symptoms can be avoided	3.28	1.15	
	Appropriateness of the vocal strain	3.28	1.25	
Workplace support		2.37	0.88	0.71
	Support for mental resources and coping at work	2.20	1.09	
	Support for promoting OW during leisure time	2.91	1.25	
	Support for promoting OW during working hours	2.14	1.14	
	Opportunities for work supervision	2.24	1.32	
Occupational health care services		3.03	1.04	0.83
	Health examinations	2.69	1.27	
	Support, advice and guidance to maintain and promote OW	2.93	1.31	
	Sufficiency of the collaboration	3.45	1.13	
	Possibility for rehabilitation	3.01	1.39	

Likert scale 1–5, 1 = total disagreement to 5 = total agreement.

Finally, to assess the associations between the WRW and its four sum variables and associations between the WRW and the overall OW variables, Pearson’s correlation coefficient (*r*) was calculated for the data and found to be normally distributed [24]. The associations between the participants’ personal, work and overall OW variables with each sum variable in the individual aspect of OW were analysed using linear regression analysis; the diagnostics of the regression models supported this method [25]. The WRW subscale’s sum variables were rated as 1 = total disagreement/very poor to 5 = total agreement/very good.

## Results

A total of 552 educators participated in this study (*n* = 1772, response rate 31%). The study participants represent voluntary respondents from the population who were working as HSCE educators either in vocational education trainee institutions (58%) or at a university of applied sciences (42%). Most of the educators were female (92%), had a permanent work contract (91%) and were married or in a relationship (84%). The participants’ mean age was 51 years (standard deviation [SD] 8.35, range 30–67) and the average work experience in HSCE as an educator was 14 years (SD 8.75, range 0–40 years); over half of the educators’ work included remote working (56%). The overall personal OW of the educators was moderate (3.19) and the overall communal OW was quite poor (2.61). Satisfaction, as regards activities to promote OW in the educators’ working community (during working hours), was quite poor (2.27) and in leisure time it was moderate (3.31) (Table 1).

Resources and mental workloads, such as backlog situations and opportunities for moments of rest, were assessed as being quite poor (2.41, SD 0.98). Resources and physical workload, such as the prevalence of vocal and musculoskeletal symptoms, scored the highest of all the sum variables and were assessed as moderate (3.49, SD 0.83). Workplace support, such as providing activities to promote OW during leisure time and working hours and having opportunities for work supervision, scored the lowest of all the subscales showing quite poor results (2.37, SD 0.88). Occupational health care services, support for the educators’ health, fitness and vigour and collaboration were assessed as being moderate (3.03, SD 1.04) (Table 2).

Pearson correlations between the WRW subscale and its sum variables were average to strong (0.62–0.80) and although there was variation between the correlations (0.18–0.55) they were still significant (Table 3) [24]. There were also positive significant (*P* < 0.01) correlations between the WRW subscale and the overall OW variables. The individual aspect of OW (WRW) correlated the strongest with the overall personal OW (*r* = 0.53) and satisfaction towards actions promoting OW in the working community (*r* = 0.58).

**Table 3.** Pearson correlations in the WRW subscale and its sum variables (*n* = 527)

	WRW	1	2	3	4
WRW	1.00				
Resources and mental workload (1)	0.75**	1.00			
Resources and physical workload (2)	0.62**	0.40**	1.00		
Workplace support (3)	0.80**	0.55**	0.35**	1.00	
Occupational health care services (4)	0.66**	0.18**	0.22**	0.36**	1.00

**Correlation is significant at the 0.01 level (two-tailed).

The linear regression analysis was used to analyse the associations between the individual aspect of OW and personal, work and overall OW variables. These independent variables explained 41–45% of the variability of the WRW’s sum variables as regards resources for mental workload and workplace support thus leaving resources for physical workload and occupational health services with lower values (Table 4). Resources for mental workload had positive associations with overall personal OW (β = 0.48, *P* < 0.001) and satisfaction towards activities promoting OW in the working community (β = 0.08, *P* = 0.022) and negative associations with the number of years working as an educator in HSCE (β = −0.02, *P* = 0.006). Workplace support had positive associations with satisfaction towards activities promoting OW in the working community (β = 0.33, *P* < 0.001) and overall personal OW (β = 0.16, *P* = 0.001) and negative associations with having underaged children in the family (β = −0.18, *P* = 0.033). Recourses and physical workload had positive associations only with satisfaction towards activities promoting OW during leisure time (β = 0.08, *P* = 0.033); occupational health care services only had positive associations with the satisfaction towards activities promoting OW in the working community (β = 0.26, *P* < 0.001).

**Table 4.** Multiple linear regression of personal, work and general OW with the WRW subscale

	Resources and mental workload			Resources and physical workload			Workplace support			Occupational health care services		
	β (SE)	*t*	CI lower/ upper	β (SE)	*t*	CI lower/ upper	β (SE)	*t*	Cl lower/ upper	β (SE)	*t*	CI lower/ upper
Constant	0.13 (0.35)	0.36	−0.57/0.82	3.07 (0.36)	8.47	2.35/3.78***	1.56 (0.33)	4.77	.92/2.20***	2.40 (0.48)	4.99	1.45/3.34***
Personal												
Age in years (continuous)	0.01 (0.01)	1.94	0.00/0.03	−0.01 (0.01)	−1.15	−0.02/0.01	−0.01 (0.01)	−1.07	−0.02/0.01	0.01 (0.01)	0.81	−0.01/0.03
In a relationship and not co-habiting/ single/widowed/ other^a^	0.10 (0.09)	1.21	−0.07/0.27	−0.04 (0.09)	−0.40	−0.21/0.14	−0.09 (0.08)	−1.17	−0.25/0.06	0.03 (0.12)	0.29	−0.20/0.26
Underage children in the family^a^	0.14 (0.09)	1.58	−0.04/0.32	−0.06 (0.09)	−0.65	−0.24/0.12	−0.18 (0.08)	−2.14	−0.35/−0.02*	0.06 (0.12)	0.50	−0.18/0.31
Caring for another person needing help (age, illness, disability)^a^	−0.03 (0.08)	−0.43	−0.18/0.12	−0.00 (0.08)	−0.01	−0.15/0.15	−0.03 (0.07)	−0.40	−0.17/0.11	0.16 (0.10)	1.51	−0.05/0.36
Work												
Remote working^a^	0.02 (0.07)	0.20	−0.13/0.16	−0.14 (0.08)	−1.89	−0.29/0.01	−0.06 (0.07)	−0.95	−0.20/0.07	−0.04 (0.10)	−0.37	−0.23/0.16
Other additional job^a^	0.03 (0.09)	0.37	−0.14/0.21	0.12 (0.09)	1.28	−0.06/0.29	−0.04 (0.08)	−0.52	−0.20/0.12	−0.04 (0.12)	−0.32	−0.28/0.20
Work experience as an educator in HSCE (continuous)	−0.02 (0.01)	−2.74	−0.03/−0.00**	−0.01 (0.01)	−0.90	−0.02/0.01	−0.00 (0.01)	−0.52	−0.01/0.01	−0.01 (0.01)	−1.20	−0.02/0.01
Overall OW (continuous)												
Personal OW	0.48 (0.05)	9.29	0.37/0.58***	0.10 (0.05)	1.91	−0.00/0.20	0.16 (0.05)	3.31	0.06/0.25***	−0.11 (0.07)	−1.50	−0.24/0.03
Communal OW	0.09 (0.06)	1.67	−0.02/0.20	0.09 (0.06)	1.56	−0.02/0.20	0.02 (0.05)	0.33	−0.08/0.12	0.02 (0.07)	0.24	−0.13/0.16
Promoting OW in the working community	0.08 (0.03)	2.30	0.01/0.15*	0.07 (0.04)	1.96	0.00/0.14	0.33 (0.03)	10.36	0.26/0.39 ***	0.26 (0.05)	5.63	0.17/0.35***
Promoting OW during leisure time	−0.05 (0.04)	−1.28	−0.12/0.03	0.08 (0.04)	2.14	0.01/0.16*	0.03 (0.04)	0.73	−0.04/0.10	0.01 (0.05)	.23	−0.09/0.11
	*R* ^2^ = 0.45			*R* ^2^ = 0.16			*R* ^2^ = 0.41			*R* ^2^ = 0.09		

β, regression coefficient; CI, 95% confidence interval; SE, standard error of regression coefficient; *t*, *t*-value.

^a^Reference category, no.

*P ≤ 0.05, **P ≤ 0.01, ***P ≤ 0.001.

## Discussion

Educators in this study assessed their individual aspect of OW as being quite poor regarding their own and the supporting resources for managing workload factors. The challenges were assessed as being in the resources for managing mental workload factors, which is in line with previous studies addressing educators’ mental strain at work [3,16]. In addition, avoiding backlog situations seemed to be the most important factor with the results for managing mental workload scoring the lowest of all the responses in this study. This has also been addressed in previous studies which have shown the need for workplace support along with personal resources to avoid mental stress from a heavy workload [3,16]. Even though the resources for managing physical workload factors had the most favourable results in this study, the educators’ work does include an extensive usage of the voice and prolonged sitting time working with computers; previous studies/a previous study has found such factors to cause physical risks [15].

Workplace support in respect of personal resources and promoting OW of the individual had the least favourable results in this study. The supporting role of the organization was found to have great importance to educators in previous studies [3,4,10]. This study found that especially workplace support towards promoting educators OW during working hours needs to be addressed. More evidence-based effective actions towards supporting personal resources (health, fitness and vigour) during working hours within this group are needed. The possibilities found for effective activities from previous intervention studies among educators in general included: expressing gratitude, voice hydration, meditation and moderate exercise in the form of walking in the school area [26]. Educators were moderately content as regards the occupational health care services provided by their working organization to support their personal resources at work. More research is needed to ascertain feasible ways for occupational health care services to support educators’ ability to cope, especially with the mental workload factors in everyday working life addressed in this study.

The associated factors concerning the individual aspect of OW, the associations of personal, work and overall OW were also under investigation. Educators having underaged children in the family were less content with the workplace support offered to promote their OW. This should lead to a consideration of the possibilities of placing the focus of their OW activities during working hours as they might have home responsibilities after work; this was also indicated in a previous study considering work–life balance [3].

HSCE educators with more work experience expressed more dissatisfaction with the resources provided to manage mental workload than those with less experience. In previous studies, experiences of high workload factors were found to be more problematic for the less experienced educators [3,16]. The job description of educators has changed in recent years, and not all the changes have favoured a sustainable workload for educators. The changes regulated by the Finnish law concerning vocational education now require a shorter time for preparing lectures and more obligations to plan individual study paths for students [27]. In addition, students are also experiencing more learning challenges, especially concerning increased remote teaching [28] which were not an issue a few years ago. This could explain why experienced educators have been affected more negatively by these changes than those who have been in the profession for less time and who are thus not able to make a comparison.

The educators’ overall personal OW had positive associations with the individual aspect of OW. This could indicate that a higher overall OW could be achieved by more investment from educator organizations and the educators themselves in personal resources to cope with stressful situations at work.

This study also has limitations that should be considered. In survey studies, a selection bias can always exist as participation is voluntary and the response rate of 31% risks the participants responding with extreme opinions on the issues. However, the study sample was quite large (~24% of the total population of educators in HSCE in Finland) and is representative of the total population concerning the participants’ mean age and gender. However, SDs within many questions were quite high indicating that diverse aspects of the individual participant’s individual experiences of OW were included.

This study was conducted within the Finnish population meaning that its generalizability to other countries must be considered carefully. This study reported one aspect (the 15-item WRW subscale) of the complete instrument used. The complete instrument includes all the aspects of the OWSS Model [9], and requires a total of 104 items to be answered; this may have prevented the educators from responding. Because the Cronbach’s alpha level of the sum variable for three items in the resources and physical workload is below the favourable level of 0.7 (0.58), the reliability of the results need to be considered [23]; however, the reliability of the total WRW subscale remained good.

## References

[1] Alker HJ , Wang ML, Pbert L, Thorsen N, Lemon SC. Impact of school staff health on work productivity in secondary schools in Massachusetts. J Sch Health 2015;85:398–404. 25877437 10.1111/josh.12266

[2] Klusmann U , Kunter M, Trautwein U et al Teachers’ occupational well-being and quality of instruction: the important role of self-regulatory patterns. J Educ Psychol 2008;100:702–715.

[3] Owens J . Life balance in nurse educators: a mixed-methods study. Nurs Educ Perspect 2017;38:182–188. 28594655 10.1097/01.NEP.0000000000000177

[4] Derby-Davis MJ . Predictors of nursing faculty’s job satisfaction and intent to stay in academe. J Prof Nurs 2014;30:19. 24503311 10.1016/j.profnurs.2013.04.001

[5] Falk N . Strategies to enhance retention and effective utilization of ageing nurse faculty. J Nurs Educ 2007;46:165–169. 17474486 10.3928/01484834-20070401-05

[6] Haryanto M . Nursing shortage: myth or fact? Orthop Nurs 2019;38:1–2. 30676566 10.1097/NOR.0000000000000535

[7] Marć M , Bartosiewicz A, Burzyńska J, Chmiel Z, Januszewicz P. A nursing shortage—a prospect of global and local policies. Int Nurs Rev 2019;66:9–16. 30039849 10.1111/inr.12473

[8] ILO. Workplace Well-being [Internet]. https://www.ilo.org/safework/areasofwork/workplace-health-promotion-and-well-being/WCMS_118396/lang--en/index.htm (11 April 2022, date last accessed).

[9] Saaranen T , Tossavainen K, Turunen H, Kiviniemi V, Vertio H. Occupational well-being of school staff members: a structural equation model. Health Educ Res 2007;22:248–260. 16880218 10.1093/her/cyl073

[10] Arian M , Soleimani M, Oghazian MB. Job satisfaction and the factors affecting satisfaction in nurse educators: a systematic review. J Prof Nurs 2018;34:389–399. 30243696 10.1016/j.profnurs.2018.07.004

[11] Baker SL , Fitzpatrick JJ, Griffin MQ. Empowerment and job satisfaction in associate degree nurse educators. Nurs Educ Perspect 2011;32:234–239. 21923003 10.5480/1536-5026-32.4.234

[12] Campbell N , Jesus S, Prapavessis H. Physical fitness. In: Gellman MD, Turner JR, eds. Encyclopedia of Behavioral Medicine. New York, NY: Springer New York, 2013; 1486–1489.

[13] Robinson P , Oades LG, Caputi P. Conceptualising and measuring mental fitness: a Delphi study. Int J Wellbeing 2015;5:53–73.

[14] Shirom A . Vigor as a positive affect at work: conceptualizing vigor, its relations with related constructs, and its antecedents and consequences. Rev Gen Psychol 2011;15(1):50–64.

[15] Sturgeon LP , Garrett-Wright D, Main E, Blackburn D, Jones MS. Nurse educators’ occupational and leisure sitting time. Workplace Health Saf 2017;65:184–187. 27856994 10.1177/2165079916665849

[16] Singh C , Cross W, Munro I, Jackson D. Occupational stress facing nurse academics—a mixed-methods systematic review. J Clin Nurs 2019;29:720–735. 10.1111/jocn.15150 31856356

[17] Rantanen J , Lehtinen S, Valenti A, Iavicoli S. A global survey on occupational health services in selected international commission on occupational health (ICOH) member countries. BMC Public Health 2017;17:787. 28982348 10.1186/s12889-017-4800-z PMC5629797

[18] Laine S , Tossavainen K, Pertel T, Lepp K, Isoaho H, Saaranen T. Occupational well-being: a structural equation model of Finnish and Estonian school. Glob J Health Sci 2018;10:79.

[19] Saaranen T , Juntunen A, Kankkunen P. Terveysalan opettajien työhyvinvointi ja sen edistäminen—työntekijän ja hänen työnsä näkökulma. Hoitotiede J Nurs Sci 2020;32:154–165.

[20] TENK. The Ethical Principles of Research with Human Participants and Ethical Review in the Human Sciences in Finland [Internet]. 2019. https://www.tenk.fi/sites/tenk.fi/files/Ihmistieteiden_eettisen_ennakkoarvioinnin_ohje_2019.pdf. (11 April 2022, date last accessed)

[21] GDPR. General Data Protection Regulation (GDPR) Compliance Guidelines [Internet]. 2018. https://gdpr.eu/. (11 April 2022, date last accessed)

[22] Howard MC . A review of exploratory factor analysis decisions and overview of current practices: what we are doing and how can we improve? Int J Hum Comput Interact 2016;32:51–62.

[23] Norman GR , Streiner DL. Biostatistics: The Bare Essentials [Internet]. 3rd edn. Hamilton, Canada: BC Decker Inc., 2008. https://books.google.fi/books?hl=fi&lr=&id=y4tWQl_8Ni8C&oi=fnd&pg=PA1&dq=+G.R.+Norman&ots=oOfJkKMrBj&sig=7qV1zMq6AmZ1lofebpL_bhyrShU&redir_esc=y#v=onepage&q=G.R.Norman&f=false. (6 March 2022, date last accessed)

[24] Mukaka MM . Statistics corner: a guide to appropriate use of correlation coefficient in medical research. Malawi Med J 2012;24:69–71. 23638278 PMC3576830

[25] Arkes J. Regression Analysis: A Practical Introduction [Internet]. Abingdon, Oxon: Routledge, 2019. http://search.ebscohost.com/login.aspx?direct=true&db=nlebk&AN=2006634&site=ehost-live. (6 March 2022, date last accessed)

[26] Rinne J , Koskinen S, Leino-Kilpi H, Saaranen T, Salminen L. Self-conductive interventions by educators aiming to promote individual occupational well-being—a systematic review. Int J Educ Res 2021;107:101755.

[27] FINLEX (531/2017). FINLEX®—Laws and Regulations—Säädökset alkuperäisinä: Laki ammatillisesta koulutuksesta 531/2017 [Internet]. Naantali, Finland: Oikeusministeriö, 2017. https://www.finlex.fi/fi/laki/alkup/2017/20170531 (11 April 2022, date last accessed).

[28] Aucejo EM , French J, Ugalde Araya MP, Zafar B. The impact of COVID-19 on student experiences and expectations: evidence from a survey. J Public Econ 2020;191:104271. 32873994 10.1016/j.jpubeco.2020.104271 PMC7451187

